# LOXL1 modulates the malignant progression of colorectal cancer by inhibiting the transcriptional activity of YAP

**DOI:** 10.1186/s12964-020-00639-1

**Published:** 2020-09-10

**Authors:** Lin Hu, Jing Wang, Yunliang Wang, Linpeng Wu, Chao Wu, Bo Mao, E. Maruthi Prasad, Yuhong Wang, Y. Eugene Chin

**Affiliations:** 1 Institutes of Biology and Medical Sciences, Soochow University, Suzhou, China; 2 Department of General surgery, The First Affiliated Hospital of Soochow University, Suzhou, China; 3 School of Biology and Basic Medical Science, Soochow University, Suzhou, China; 4 Department of Cell Biology and Genetics, Shenzhen key of Laboratory of Translational medicine of Tumor, Shenzhen University Health science center, Shenzhen, China; 5 Department of Pathology, The First Affiliated Hospital of Soochow University, Suzhou, China

**Keywords:** Colorectal cancer, LOXL1, Tumorigenesis, Yes-associated protein

## Abstract

**Background:**

LOX-like 1 (LOXL1) is a lysyl oxidase, and emerging evidence has revealed its effect on malignant cancer progression. However, its role in colorectal cancer (CRC) and the underlying molecular mechanisms have not yet been elucidated.

**Methods:**

LOXL1 expression in colorectal cancer was detected by immunohistochemistry, western blotting and real-time PCR. In vitro, colony formation, wound healing, migration and invasion assays were performed to investigate the effects of LOXL1 on cell proliferation, migration and invasion. In vivo, metastasis models and mouse xenografts were used to assess tumorigenicity and metastasis ability. Molecular biology experiments were utilized to reveal the underlying mechanisms by which LOXL1 modulates the Hippo pathway.

**Results:**

LOXL1 was highly expressed in normal colon tissues compared with cancer tissues. In vitro*,* silencing LOXL1 in CRC cell lines dramatically enhanced migration, invasion, and colony formation, while overexpression of LOXL1 exerted the opposite effects. The results of the in vivo experiments demonstrated that the overexpression of LOXL1 in CRC cell lines drastically inhibited metastatic progression and tumour growth. Mechanistically, LOXL1 inhibited the transcriptional activity of Yes-associated protein (YAP) by interacting with MST1/2 and increasing the phosphorylation of MST1/2.

**Conclusions:**

LOXL1 may function as an important tumour suppressor in regulating tumour growth, invasion and metastasis via negative regulation of YAP activity.

**Video abstract**

**Graphical abstract:**

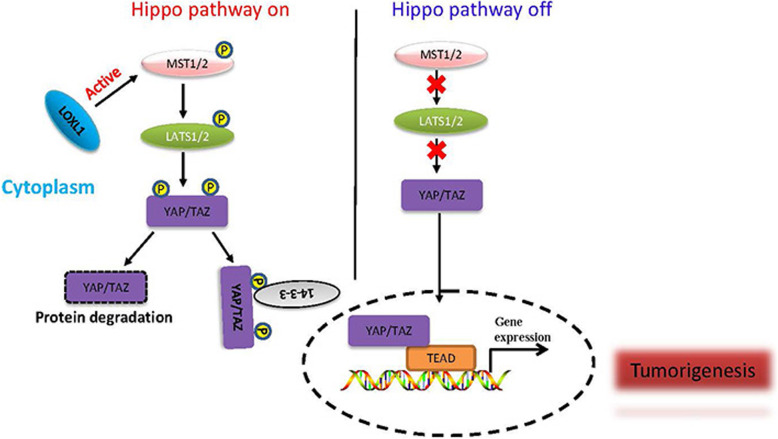

## Background

Colorectal cancer (CRC) is ranked as the third most frequently diagnosed cancer and the second leading cause of cancer-related death worldwide [1]. Moreover, the five-year relative survival rate for surgical patients in the late stages of colon cancer is only approximately 10% [2], and over 50% of patients with colon cancer are clinically diagnosed at the late stages [3]. Invasion and distant metastasis of the tumours are considered to be the reasons leading most frequently to the mortalities associated with CRC. However, the mechanisms underlying this malignant progression are not fully understood. Therefore, investigation of the associated mechanisms is very important for developing strategies to treat patients with CRC.

The Hippo pathway is an evolutionarily conserved tumour suppressor pathway best known for its roles in modulating organ size, tissue homeostasis, and tumour progression [4]. Recent studies have revealed that dysregulation of the Hippo signalling pathway is associated with the progression of CRC [5–7]. In mammals, MST1/2, SAV1, LATS1/2, and MOB1 have been reported as the core kinase components [8]. When Hippo signalling is activated, MST1/2 utilizes SAV1 and other scaffolds to phosphorylate and activate LATS1/2, which results in the phosphorylation of YAP at multiple sites, and its transcriptional activity is inhibited through cytoplasmic confiscation or ubiquitin-mediated degradation. While signalling is inhibited, unphosphorylated YAP and TAZ are transferred to the nucleus, where they are combined with TEAD. Then, the YAP/TAZ/TEAD complex induces the expression of target genes associated with Hippo-YAP, which include *CDC20*, *CDX2A*, *CTGF*, and *CYR61* [9]. Previous reports have suggested that Hippo signalling plays a critical role in the growth, invasion and metastasis of colon tumours [10, 11]. Therefore, understanding the regulatory mechanism of Hippo-YAP signalling is essential to determine the progression of CRC.

The lysyl oxidase (LOX) family of copper-dependent ε-amine lysine oxidases was first identified in mammalian cells and yeast [12]; this family was found to contain five identified paralogues, which are as follows: LOX, LOX-like 1 (LOXL1), LOX-like 2 (LOXL2), LOX-like 3 (LOXL3), and LOX-like 4 (LOXL4). LOX enzymes catalyse the oxidative deamination of ε-amino groups of lysine and hydroxylysine residues on collagen and elastin, generating reactive aldehydes. The aldehydes can condense with neighboring aldehydes or ε-amino groups to form higher-order cross-linkages [13]. Furthermore, reactions such as the Amadori Rearrangement can form extremely complex crosslinks [14]. The catalytic domain of LOX enzymes contains one copper binding motif and the functional quinone group, which has been identified as lysyl tyrosylquinone (LTQ) derived from posttranslational cross-linkage between a specific lysine and a specific tyrosine [15]. Contente, et al. (1999) reported that LOX is a tumour suppressor for the first time [16]. Csiszar et al. (2002) also reported that LOX could be considered a tumour suppressor in CRC [17]. Furthermore, Wu et al. (2007) reported that LOXL1 suppresses the growth of bladder cancer [18]. However, Loxl1 is upregulated in Lkb1-deficient mice with enhanced metastasis [19]. LOXL1 expression is associated with chemotherapy resistance in pancreatic ductal carcinoma and non-small cell lung cancer (NSCLC) [20, 21]. In addition, LOXL1 is regulated by integrin α11 and promotes NSCLC progression [22, 23]. To date, few studies on the role of LOXL1 in the progression of CRC are available. In our previous studies, it has been reported that LOXL3 lacking the signal peptide (SP) can function as a deacetylase in the nuclei facilitating Th17 cell differentiation through the regulation of STAT3 deacetylation [24]. Hence, our aim was to determine the exact effects and mechanisms underlying the involvement of LOXL1 in CRC.

Here, we demonstrated that the overexpression of LOXL1 repressed cell migration, invasion, and tumorigenesis in vitro and in vivo. In contrast, knockdown of LOXL1 in CRC cells resulted in the opposite effect. The results of the luciferase reporter assays revealed that LOXL1 inhibited the transcriptional activity of YAP. Moreover, SP deletion in LOXL1 strongly inhibited cellular secretions and the activity of YAP. We also determined that LOXL1 induced the activity of MST1/2 kinase. Therefore, we hypothesized that intracellular LOXL1 inhibits the malignancy of CRC through a p-YAP-dependent signalling pathway. Consistent with our hypothesis, the overexpression of LOXL1 with SP deletion significantly suppressed the migration and invasive abilities of CRC cells. Overall, our results revealed the novel molecular mechanisms by which LOXL1 inhibits the malignant progression of CRC in a YAP-dependent manner.

## Methods

### Immunohistochemistry (IHC)

The LOXL1 expression levels were assessed using IHC on the paired paraffin-preserved tissue sections of 30 CRC patients and 15 CRC patients with liver metastasis. Immunohistochemistry was performed on 2 μm sections using the BenchMark ULTRA automated stainer (Ventana Medical Systems, Inc., Tucson, Arizona, USA) in accordance with the manufacturer’s protocols. Primary LOXL1 antibody was obtained from Sigma (HPA042111, anti-LOXL1 diluted 1:50). Each specimen was scored according to the proportion of positive cancer cells as follows: 1, 0–25%; 2, 25–50%; 3, 50–75%; and 4, > 75%. Specimens were also scored according to the staining intensity of cancer cells as follows: 0, negative; 1, light yellow; 2, dark yellow; 3, brown. The IHC staining score was calculated by multiplying the proportion of positive cancer cells by the staining intensity of cancer cells. The staining results were evaluated by two independent pathologists who had at least 5 years working experience.All samples were obtained with approval from the Institutional Ethics Committee of The First Affiliated Hospital of Soochow University (authorisation number ECSU-2019000212).

### Cell culture

Human colorectal cancer cell lines, such as DLD1, HCT116, HCT8, HT29, LoVo, SW480, SW620, and RKO, were purchased from American Type Culture Collection (ATCC). All CRC cell lines were maintained in RPMI 1640 supplemented with 10% foetal bovine serum and 1% antibiotic (penicillin and streptomycin) at 37 °C in an atmosphere of 5% CO_2_ and 95% air.

### Lentiviral vector construction and packaging

Lentiviral constructs of LOXL1 encoding pLenti-EF1a-FH-CMV-GFP-P2A-puromycin were prepared as described previously [25]. Lentivirus expressing *LOXL1* was produced in HEK293T cells and then packaged using pMD2.G and psPAX2. The HCT8 and SW480 cell lines were infected with the viral supernatant using 8 μg/mL polybrene (TR-1003-G, Sigma), and the infected cells were incubated for 48 h. Single colonies were obtained through puromycin selection (8 μg/mL), which were detected using western blotting.

### Wound healing assay

First, 1 × 10^6^ cells were cultured in six-well plates and incubated for 24 h. The cultured cells were rinsed thrice using phosphate buffered saline (PBS), and three wounds (scratches) were created in parallel using a sterile 200-μL pipette tip. The wells were washed thrice with PBS to discard any floating cells. Representative images of their migration were captured immediately using a microscope (Nikon, Eclipse Ti-S) 24 h and 48 h after scratching.

### In vitro Transwell migration and invasion

Cell migration and invasion experiments were performed using 24-well plates with 8 μm-polycarbonate filter inserts (#3422, Corning). HCT8-N/HCT8-LOXL1, SW480-N/SW480-LOXL1, HT29-N/HT29-LOXL1 (knockdown), and RKO-N/RKO-LOXL1 (knockdown) cells were seeded at densities of 2 × 10^5^ cells/200 μL and 1 × 10^5^ cells/200 μL per well, respectively, in serum-free RPMI 1640. All cells were either uncoated or Matrigel-coated (#354234, Biocoat) and incubated in chambers containing 600 μL of RPMI 1640 with 10% foetal serum as a chemoattractant. The cells were imaged, and their migration and invasion were captured using a microscope (Nikon, Eclipse Ti-S). The migrating and invading cells were eluted using acetic acid and quantified by measuring their absorbance at 570 nm. All experiments were performed thrice independently.

### Plate colony formation assay

HCT8-N/HCT8-LOXL1, SW480-N/SW480-LOXL1, RKO-N/RKO-LOXL1 (knockdown), and HT29-N/HT29-LOXL1 (knockdown) cells were independently seeded in six-well plates at densities of 5000 cells/well at 37 °C. The medium, RPMI 1640 containing 10% foetal bovine serum, was changed every alternate day. After 10 days, the cells forming colonies were immersed in 4% paraformaldehyde for 20–30 min, stained using crystal violet for 2 h, and rinsed thrice with PBS to remove the excess crystal violet. Finally, images were captured using a microscope, and the number of colony-forming units was counted.

### Luciferase reporter assay

The 3× GTIIC promoter was subcloned into the *Xho*I/*Hind*III site of the pGL4.2 vector (Promega). HEK293T cells were transiently co-transfected with the pGL4.2–3× GTIIC, pcDNA3.1-YAP, and LOXL1/LOXL1 ∆SP/LOXL1 mutants. The pRL-TK vector was co-transfected in each experimental well as an internal control. After 24 h of transfection, the cells were collected and analysed using the Dual-Luciferase Reporter Assay Kit (E1910, Promega).

### Immunoprecipitation and western blotting

HEK293T cells were transfected with *FL LOXL1* and its mutants. After 24 h, the medium was collected and centrifuged at 1000 *g* for 5 min. The supernatant was subjected to immunoprecipitation using M2-conjugated magnetic beads (M8823, Sigma) by rotating for 4 h at 4 °C. The immunoprecipitates were washed three times using PBS and subjected to western blot analysis. Additionally, the cells were lysed using a lysis buffer (20 mM Tris-HCl, pH 7.4, 150 mM NaCl, 0.5% NP-40, 10% glycerol, 1 mM DTT, and the complete protease inhibitor cocktail) for 10 min on ice and centrifuged at 20,000 *g* for 30 min. The cell lysates were analysed by subjecting them to SDS-PAGE and immunoblotting with antibodies as indicated in the figures.

### Total RNA isolation and quantitative reverse transcription-polymerase chain reaction (qRT-PCR)

Expression of the genes *CYR61*, *CDC20*, *CDX2A*, and *CTGF* was detected using qRT-PCR and normalized to that of GAPDH. Total RNA was extracted using TRIzol (DP424, TIANGEN), according to the manufacturer’s protocol. Total RNA (1 μg) was reverse transcribed using the PrimeScript RT reagent Kit (RR037A, TaKaRa). SYBR green (B21202, Bimake) and an ABI Step One Plus real-time PCR system (Applied Biosystems) were used to conduct qRT-PCR. The primers used in this study are listed in Table 1.

**Table 1:** Primers used for real-time quantitative PCR (qPCR)

Target mRNA	Sequences 5′—3′
*GAPDH*	
F	5′- GGAGCGAGATCCCTCCAAAAT-3’
R	5′- GGCTGTTGTCATACTTCTCATGG-3
*CDX2*	
F	5′-CCAATGACAACGCCTCCTG-3’
R	5′-TGGTGCAGCCAGAAAGCTC-3’
*CTGF*	
F	5′-AAAAGTGCATCCGTACTCCCA-3’
R	5′-CCGTCGGTACATACTCCACAG- 3’
*CYR61*	
F	5′- AGCCTCGCATCCTATACAACC- 3’
R	5′- TTCTTTCACAAGGCGGCACTC-3’
*CDC20*	
F	5′-GACCACTCCTAGCAAACCTGG-3’
R	5′- GGGCGTCTGGCTGTTTTCA-3’

### Cell apoptosis analysis

To analyse the fraction of apoptotic cells, HCT8-N/HCT8-LOXL1 and SW480-N/SW480-LOXL1 cells were assessed with an annexin V-APC/7-AAD apoptosis detection kit (KA3808, Abnoya). Briefly, each sample containing 1 × 10^5^ cells was washed twice with cold phosphate-buffered saline (PBS) and then resuspended cells in 100 μL 1 × binding buffer. Then, 5 μl of 7-AAD and 5 μl of APC annexin V were added to each sample. The cells were incubated in the dark for 15 min at room temperature. Approximately 10,000 cells/sample were analysed by flow cytometry (Becton, Dickinson and Company, FACS Canto II).

To analyse the apoptotic cells in xenografted tumours, the samples were fixed in 10% formalin and were paraffin-embedded in the Pathology Facility of First Affiliated Hospital of Soochow University. TUNEL analysis was conducted by a commercial company (Wuhan Servicebio Technology CO., Ltd).

### Immunofluorescence

HCT8 cells grown on slides were washed with PBS, fixed with 4% paraformaldehyde (PFA) for 15 min, and permeabilized in 0.1% Triton X-100 in PBS. Fixed cells were incubated with anti-YAP antibody (#1407S, CST) overnight at 4 °C. Alexa fluorescence 546-labelled secondary antibody was applied for 60 min at room temperature (Invitrogen Life Technologies). All the samples were then stained with 4′,6-diamidino-2-phenylindole (DAPI). All images were collected using a Nikon A1 confocal microscope.

### Animal experiments

To carry out the xenograft tumorigenesis assays, 1 × 10^7^ HCT8-N/HCT8-LOXL1 cells were subcutaneously injected into 4-week-old male nude BALB/c mice. The tumour sizes were monitored every 3 d, and their volumes were determined using the following formula: volume (mm^3^) = (length×width^2^)/2. Subsequently, they were subjected to H-E staining and immunohistochemistry (IHC).

To carry out the tail vein metastasis assay, cells were injected into the lateral tail veins of 4 week-old male nude BALB/c mice. Eight weeks later, the mice were anesthetized using nembutal (pentobarbital, TRC). The mice were sacrificed and examined at necropsy for the presence of metastases. Their lungs, livers, and bones were fixed in formalin. Subsequently, the samples were subjected to H-E staining and IHC.

To conduct the liver metastasis assay, cells were harvested using 0.25% trypsin, washed thrice with PBS, and suspended in PBS at a final concentration of 1.5 × 10^7^ cells/mL. The 6-week-old BALB/c nude mice were anesthetized through an intraperitoneal injection of nembutal at a dose of 75 mg/kg. Then, a small incision, approximately 10 mm in length, was made through the skin over the spleen. Using a 27 gauge needle, 100 μL of the tumour cell suspension was slowly injected into the spleen, after which it was placed back in the abdominal cavity. The incision was closed through simple continuous suturing. The mice were sacrificed after 20 d and liver metastasis was confirmed pathologically [26].

All animal experiments were approved by the Animal Care and Use Committee as well as the Ethical Committee of Soochow University (SYXK2017–0043). All surgeries were performed under sodium pentobarbital anaesthesia with minimum fear, anxiety and pain.

### Statistical analysis

The data obtained were statistically analysed using SPSS (version 20.0; IBM, New York) and represented as the mean ± SD. A *t*-test (for two groups) was used to determine differences between the groups, which were considered statistically significant at *P* < 0.05.

## Results

### LOXL1 expression is significantly downregulated in CRC and CRC liver metastasis tissues

**Fig. 1:**
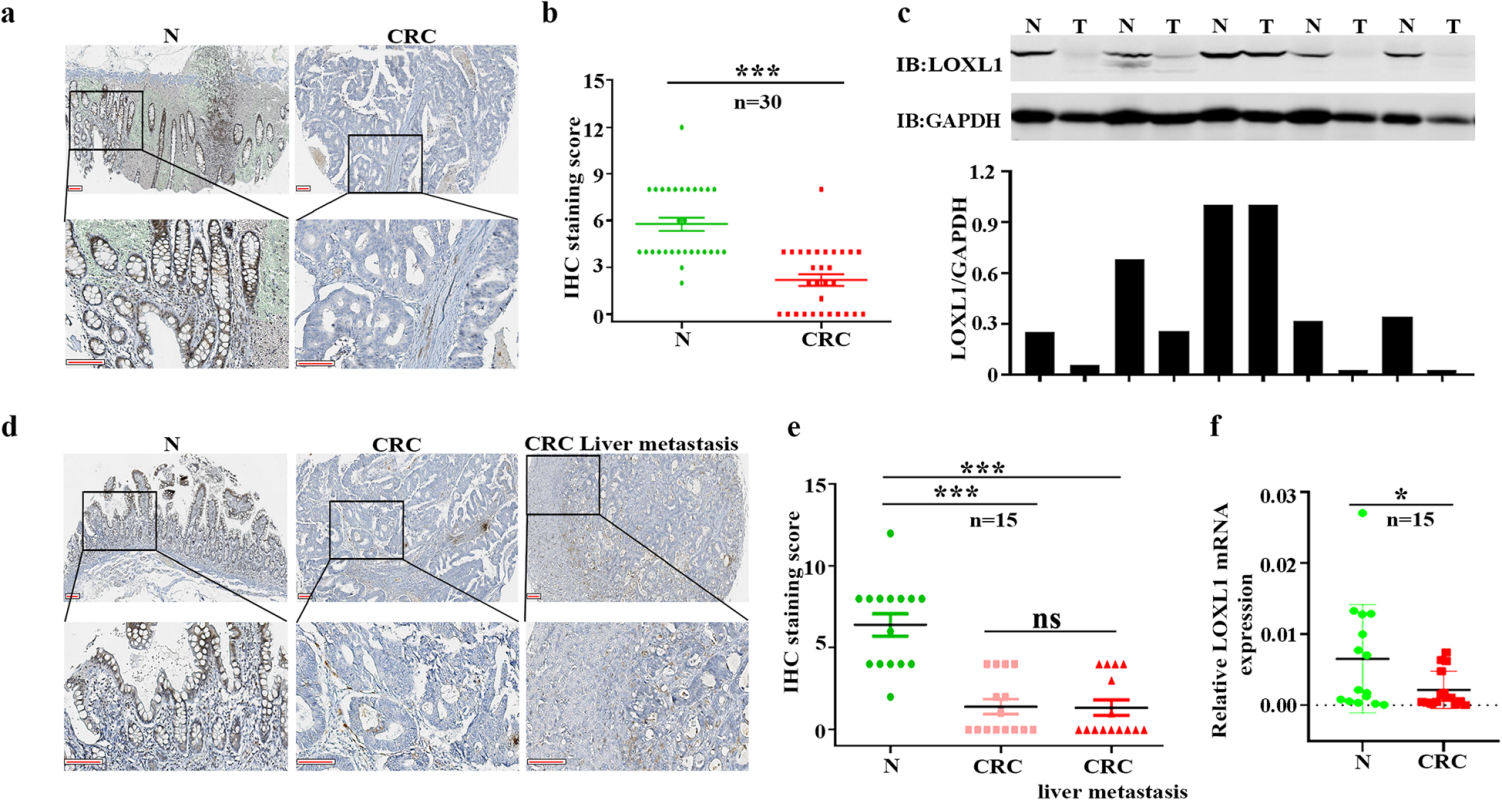
LOXL1 expression is significantly downregulated in CRC and CRC liver metastasis tissues. **a** IHC staining performed using an antibody against LOXL1 and representative photographs of LOXL1 in normal (N) and CRC tissues. Scale bar: 100 μm. **b** IHC staining score of LOXL1 in the 30 pairs of CRC patients. Data are presented as the mean ± SD; statistical significance was assessed by paired t-test. ***P < 0.001 *n* = 30. **c** Western blot analysis was performed using an antibody against LOXL1 in 5 pairs of CRC patients samples (upper panel) and protein band intensities were measured by Image J software and normalized to GAPDH (lower panel). **d** IHC staining performed using an antibody against LOXL1 and representative photographs of LOXL1 in 15 pairs of CRC patients with liver metastasis. Scale bar: 100 μm. **e** IHC staining score of LOXL1 in the 15 pairs of samples from CRC patients with liver metastasis. Data are presented as the mean ± SD; statistical significance was assessed by paired t-test. ***P < 0.001 *n* = 15. **f** qRT-PCR analysis of LOXL1 mRNA expression in 15 pairs of CRC patient samples. Data are shown as the mean ± SD of triplicate independent sets of experiments; statistical significance was assessed by paired t-test. *P < 0.05 *n* = 15

To illustrate the expression pattern of LOXL1 in CRC, we evaluated the protein expression level of LOXL1 in 30 paired CRC and adjacent normal tissues by immunohistochemistry (IHC). We observed a significantly lower expression of LOXL1 in CRC samples than in adjacent non-tumour samples (Fig. 1a), and the difference in IHC staining scores was statistically significant (*P* < 0.001) (Fig. 1b). Consistently, western blot analysis of 5 paried CRC and adjacent normal tissues also revealed that LOXL1 expression was dramatically lower in CRC than in paired normal tissues (Fig. 1c). In another 15 independent CRC patients with liver metastasis, we also observed a significantly lower expression of LOXL1 in CRC and CRC with liver metastasis tissues than in normal colorectal tissues (Fig. 1d), and the difference in IHC staining scores was statistically significant (*P* < 0.001) (Fig. 1e). We further examined the mRNA expression of LOXL1 in 15 pairs of CRC and adjacent normal tissues; the result also demonstrated that LOXL1 is downregulaed in CRC, and the result was statistically significant (*P* < 0.05) (Fig. 1f). All of these data indicate that LOXL1 is expressed at lower levels in CRC and CRC with liver metastasis than in normal tissues.

### Overexpression of LOXL1 decreases the migration and invasion abilities of CRC cells in vitro

**Fig. 2:**
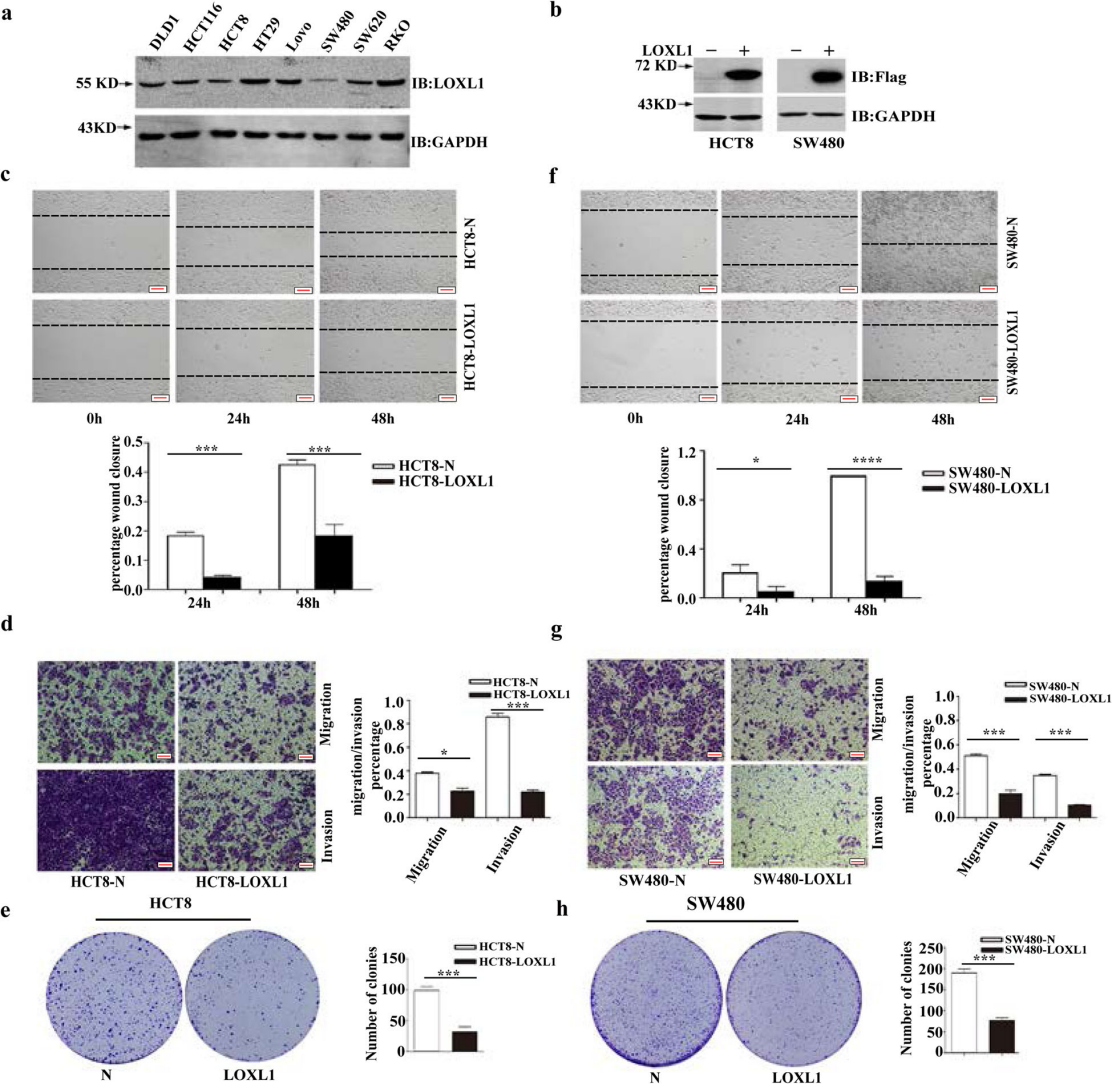
Effect of LOXL1 expression on the migration and invasion of HCT8 and SW480 cells in vitro*.* **a** Western blot analysis demonstrating the expression of LOXL1 in CRC cell lines. Total GAPDH was used as a loading control. **b** Western blot analysis of HCT8/SW480 cells stably transfected with LOXL1 overexpression lentiviruses or control lentiviruses. Total GAPDH served as a loading control. **c** Wound healing analysis: effect of the overexpression of LOXL1 in stable HCT8 cells at 0 h, 24 h, and 48 h (upper panel) and calculation of the percentage of wound healing (lower panel). Data are shown as the mean ± SD of triplicate independent sets of experiments; statistical significance was assessed by unpaired t-test. ***P < 0.001. Scale bar: 100 μm. **d** Transwell migration and Matrigel invasion assays using stable HCT8-LOXL1 and HCT8-N cells (left panel) and calculation of the rate of migration/invasion in relevant stable HCT8 cells (right panel). Data are shown as the mean ± SD of triplicate independent sets of experiments; statistical significance was assessed by unpaired t-test. *P < 0.05, ***P < 0.001. Scale bar: 100 μm. **e** A colony formation assay was performed in HCT8 cells with or without LOXL1 overexpression. Left panel: representative images, right panel: quantification analysis. Data are shown as the mean ± SD of triplicate independent sets of experiments; statistical significance was assessed by unpaired t-test. ***P < 0.001. **f** Wound healing analysis to determine the effect of LOXL1 overexpression in stable SW480 cells at 0 h, 24 h, and 48 h (upper panel) and calculation of their wound healing percentages (lower panel). Data are shown as the mean ± SD of triplicate independent sets of experiments; statistical significance was assessed by unpaired t-test. *P < 0.05, ****P < 0.0001. Scale bar: 100 μm. **g** Transwell migration and Matrigel invasion assays using stable SW480-LOXL1 and SW480-N cell lines (left panel) and calculation of the rate of migration/invasion in corresponding stable SW480 cells (right panel). Data are shown as the mean ± SD of triplicate independent sets of experiments; statistical significance was assessed by unpaired t-test. ***P < 0.001. Scale bar: 100 μm. **h** A colony formation assay was performed using SW480 cells with or without LOXL1 overexpression. Left panel: representative images, right panel: quantification analysis. Data from independent experiments are presented as the mean ± SD. Statistical significance was assessed by unpaired t-test; ***P < 0.001.

We evaluated the expression of LOXL1 in CRC cell lines such as DLD1, HCT116, HCT8, HT29, LoVo, SW480, SW620, and RKO. Because lower levels of LOXL1 are expressed in HCT8 and SW480 cells compared to other CRC cell lines, these cell lines were selected for conducting the experiments (Fig. 2a). Then, lentiviral constructs expressing LOXL1 were used to overexpress LOXL1 in HCT8 and SW480 cell lines to investigate its role in the malignant progression of CRC. The transfection efficiency was analysed through western blotting and the results revealed that LOXL1 was markedly overexpressed in HCT8 and SW480 cells (Fig. 2b). The induction of cancer metastasis is dependent on the migration and invasive properties of cancerous cells. The wound healing assay is a well-established methodology, that is evaluated to determine their migration potential. We conducted a wound healing assay to investigate the effect of LOXL1 on the migration of CRC cells in models involving HCT8 and SW480 cells. The results revealed that wound healing was slower in the presence of LOXL1 than in the negative control group (Fig. 2c and f). To assess the contribution of LOXL1 to the development of migratory and invasive phenotypes of CRC cells, migration and invasion experiments were conducted using both HCT8 and SW480 cells, in which the expression of LOXL1 and the control vector were found to be stable. The data indicated that the overexpression of LOXL1 significantly decreased the migration and invasion of HCT8 and SW480 cells (Fig. 2d and g). Colony formation assays were carried out to investigate the effect of LOXL1 on the proliferation of CRC cells. Overexpression of LOXL1 was found to significantly inhibit the colony formation ability (Fig. 2e and h) of HCT8 and SW480 cells compared to that of the control. Furthermore, the proliferation ability of HCT8/SW480 LOXL1 stable overexpressed cells was also measured by CCK8 assay (Fig. S1a). These results showed that LOXL1 inhibits the proliferation of CRC cells. Next, we investigated whether the above-observed inhibition of proliferation was due to increased cell death, and we detected the effect of overexpression of LOXL1 on apoptosis in HCT8/SW480 cells using an apoptosis detection kit. To our surprise, overexpression of LOXL1 did not affect early or late apoptosis in HCT8/SW480 cells (Fig. S1b). Collectively, these observations suggest that LOXL1 is a negative regulator of migration, invasion, and tumorigenesis in CRC cells.

### Knockdown of *LOXL1* enhances the migratory and invasive abilities of CRC cells in vitro

**Fig. 3:**
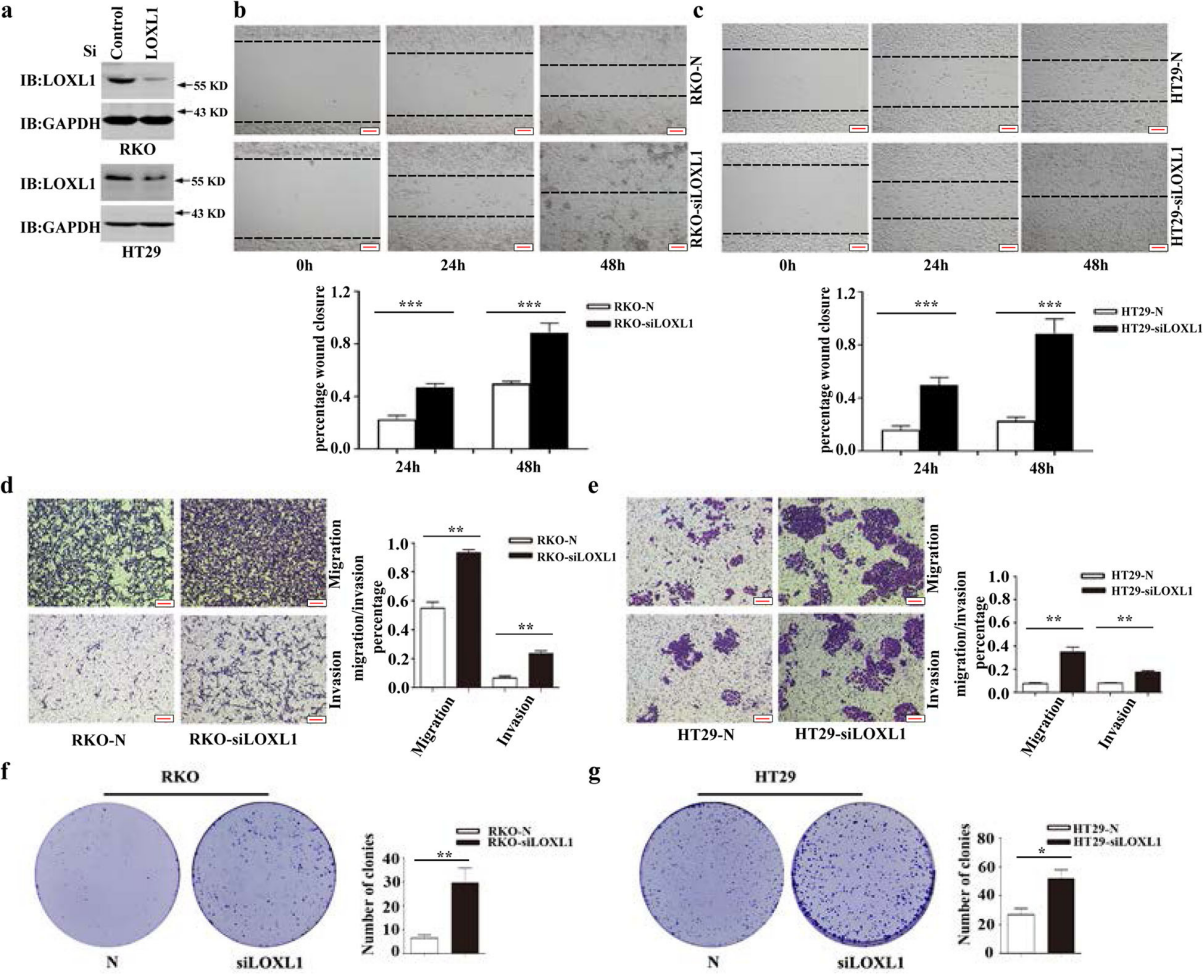
Knockdown of *LOXL1* in RKO and HT29 cells promotes their migration and invasion in vitro*.* **a** Western blot analysis of LOXL1 knockdown in RKO and HT29 cells expressing LOXL1-targeting or control siRNA. Total GAPDH was used as a loading control. **b-c** Wound healing analysis upon *LOXL1* knockdown in RKO and HT29 cells transiently transfected with LOXL1-targeting or scramble siRNA at 0 h, 24 h, and 48 h. Representative images (upper panel) and quantification (lower panel) are shown as indicated. Data from three independent experiments are presented as the mean ± SD. Statistical significance was assessed by unpaired t-test; ***P < 0.001. Scale bar: 100 μm. **d-e** Transwell migration and Matrigel invasion assays were performed in LOXL1-knockdown and control cells. Representative images (left panel) and quantification (right panel) are shown as indicated. Data from three independent experiments are presented as the mean ± SD. Statistical significance was assessed by unpaired t-test; **P < 0.01. Scale bar: 100 μm. **f-g** A colony formation assay was performed using RKO and HT29 cells transfected with LOXL1-targeting or scramble siRNA. Left panel: representative images, right panel: quantification analysis. Data from independent experiments are presented as the mean ± SD. Statistical significance was assessed by unpaired t-test; *P < 0.05, **P < 0.01

Next, knockdown of *LOXL1* in RKO and HT29 cells expressing high amounts of endogenous LOXL1 was carried out, as described in Fig. 2a, followed by western blot analysis, which enabled determination of the transient transfection efficiency of siRNA (Fig. 3a). A wound healing assay was performed to explore the effects of LOXL1 on the migration of RKO and HT29 cells. The results revealed that wound healing was highly regulated in the absence of LOXL1, and the migration potential of these cells was high compared to that of the control (Fig. 3b and c). Using Transwell migration and invasion assays, we demonstrated that the knockdown of *LOXL1* significantly increased the migration and invasion of RKO and HT29 cells compared to negative control cells (Fig. 3d and e). We also carried out a colony formation assay in the absence of LOXL1 to determine its effect on the tumorigenesis of RKO and HT29 cells. The results revealed that a reduction in the expression levels of LOXL1 allowed a significant increase in the colony formation ability compared to that observed in the control cells (Fig. 3f and g). The proliferation ability of RKO/HT29 LOXL1 knockdown cells was also measured by CCK8 assay (Fig. S2). These results showed that LOXL1 silencing promoted the proliferation of CRC cells. Taken together, these observations (Figs. 2 and 3) suggest that LOXL1 acts as a tumour suppressor and facilitates the migration, invasion, and tumourigenesis of CRC cells.

### Intracellular LOXL1 inhibits the transcriptional activity of YAP

**Fig. 4:**
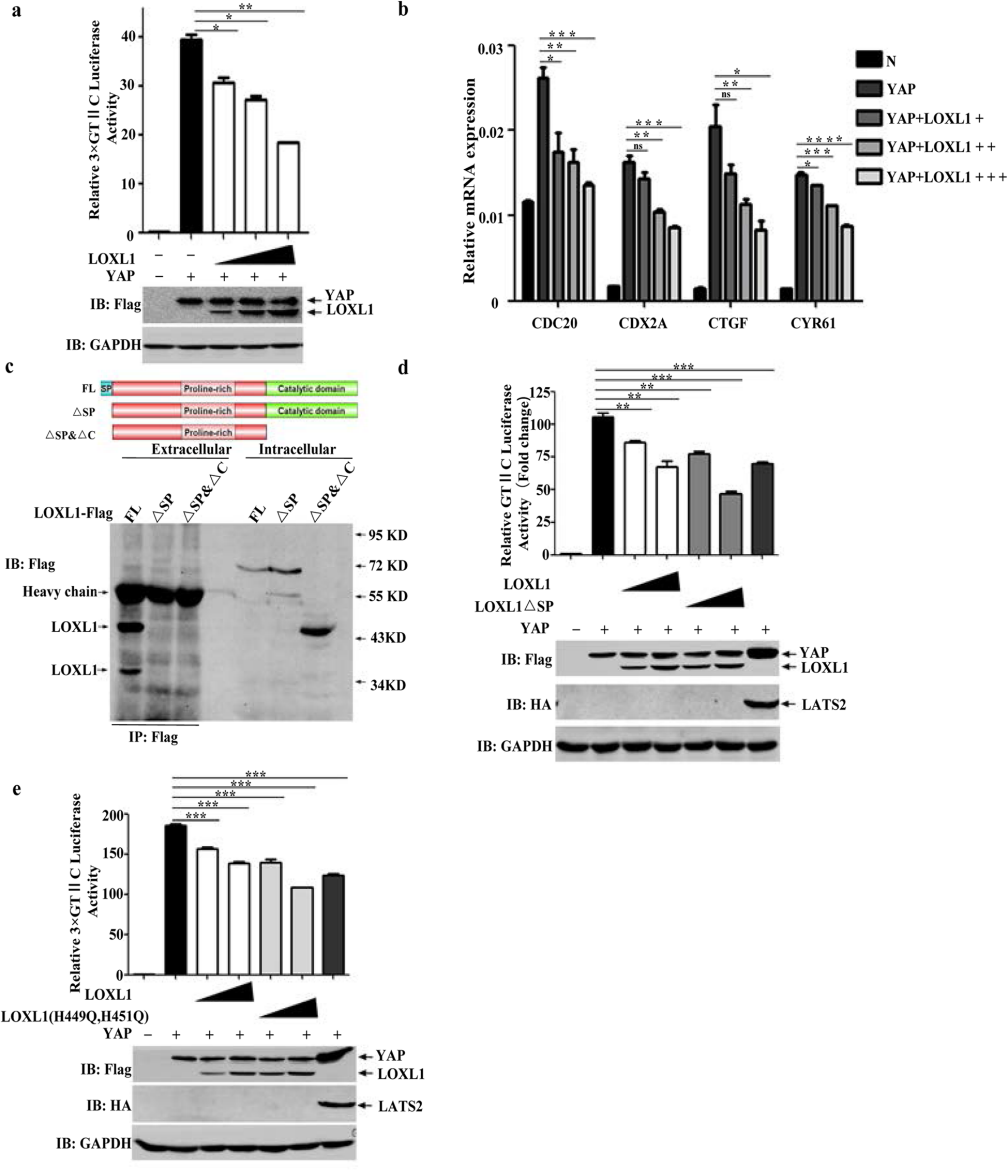
Intracellular LOXL1 inhibits the transcriptional activity of *YAP.* **a** LOXL1 and YAP constructs alone or combined, as indicated, were transfected into HEK293T cells together with a 3× GTIIC luciferase reporter. The results shown were normalized for transfection efficiency. The cell lysates were immunoblotted with the indicated antibodies (lower panel). Data are shown as the mean ± SD of triplicate independent sets of experiments; statistical significance was assessed by unpaired t-test. *P < 0.05, **P < 0.01. **b** LOXL1 and YAP alone or combined as indicated were transfected into HEK293T cells for 24 h. Total RNA was prepared, and qRT-PCR was conducted to measure the induction of *CDC20*, *CDX2A*, *CTGF*, and *CYR61*. Data are shown as the mean ± SD of triplicate independent sets of experiments; statistical significance was assessed by unpaired t-test. ns; non-significant, *P < 0.05, **P < 0.01, ***P < 0.001, ****P < 0.0001. **c** LOXL1 or its truncations were transfected into HEK293T cells for 24 h. Flag immunoprecipitates prepared from DMEM and whole cell lysates were analysed by western blotting with a Flag antibody. **d** In HEK293T cells, LOXL1, LOXL1 △SP, LATS2 and YAP alone or combined as indicated were tested for YAP binding promoter 3× GTIIC luciferase activity induction. The cellular extracts were immunoblotted with the indicated antibodies (lower panel). Data are shown as the mean ± SD of triplicate independent sets of experiments; statistical significance was assessed by unpaired t-test. **P < 0.01, ***P < 0.001. **e** In HEK293T cells, LOXL1, LOXL1 mutant, LATS2 and YAP alone or combined as indicated were tested for YAP binding promoter 3× GTIIC luciferase activity induction. The cell lysates were immunoblotted with the indicated antibodies (lower panel). The data are shown as the mean ± SD of triplicate independent sets of experiments; ***P < 0.001

To explore the signalling pathway by which LOXL1 exerted its antitumour effects, we performed dual luciferase reporter assays to measure diverse signalling pathways (Fig. S3a). We found that LOXL1 negatively regulated the activity of YAP. The Hippo-YAP signalling pathway is one of the most important pathways involved in epithelial-mesenchymal transition (EMT) and metastasis [27–29]. To verify this mechanism, we measured the transcriptional activity of YAP by co-transfecting HEK293T cells with the 3× GTIIC luciferase reporter [30–32] and different dosages of LOXL1 constructs. The results of the luciferase reporter assay revealed that the activity of the 3× GTIIC was drastically suppressed by LOXL1 in a dose-dependent manner. The results of the western blot analysis suggested that the overexpression of LOXL1 did not change the total expression levels of YAP (Fig. 4a). Additionally, mRNA studies revealed that LOXL1 repressed the expression of *CDC20*, *CDX2A*, *CTGF*, and *CYR61* in a dose-dependent manner after its transient expression in HEK293T cells, and these genes have been reported as genes downstream of YAP. (Fig. 4b). Immunofluorescence showed that YAP was localized to both the cytoplasm and the nucleus, but YAP was mainly present in the cytoplasm in LOXL1-overexpressing HCT8 cells (Fig. S3b). LOXL1 contains a signal peptide, pro-sequence, and proline-rich and catalytic domains. Among these domains, the signal peptide and catalytic domains are responsible for the secretion of LOXL1 and mediating its enzyme activity, respectively. We identified the domain of LOXL1 responsible for the inhibition of YAP. We constructed various expression plasmids, which included those with deleted signal peptide (LOXL1 ∆SP), deleted signal peptide and the C terminus (LOXL1 ∆SP & ∆C), and mutated amino acids (H449 to Q449, H451 to Q451) to facilitate the loss of the enzyme activity of LOXL1. Initially, the supernatant of cultured cells was harvested and used to conduct the immunoprecipitation assay. The results demonstrated that LOXL1 FL was extracellularly secreted in significant amounts in the form of two short isoforms. However, their presence was not detected in the LOXL1 ∆SP and LOXL1 ∆SP & ∆C groups (Fig. 4c). Based on these observations, we further determined that LOXL1 ∆SP inhibited the activities of the 3× GTIIC reporters in a dose-dependent manner; the effect of LOXL1 ∆SP was more potent than that of LOXL1 FL, but the expression of total YAP was not affected; LAST2 was used as a positive control (Fig. 4d). However, in the absence of its enzyme activity, LOXL1 still inhibited the transcriptional activity of YAP (Fig. 4e). Collectively, these results indicated that LOXL1 negatively regulated the transcriptional activity of YAP, while deletion of SP in LOXL1 resulted in its intracellular retention and demonstrated stronger inhibition. Furthermore, it was determined that this function did not depend on the enzyme activity of the lysine oxidases of LOXL1.

### LOXL1 activates the hippo pathway through interaction with MST1/2

**Fig. 5:**
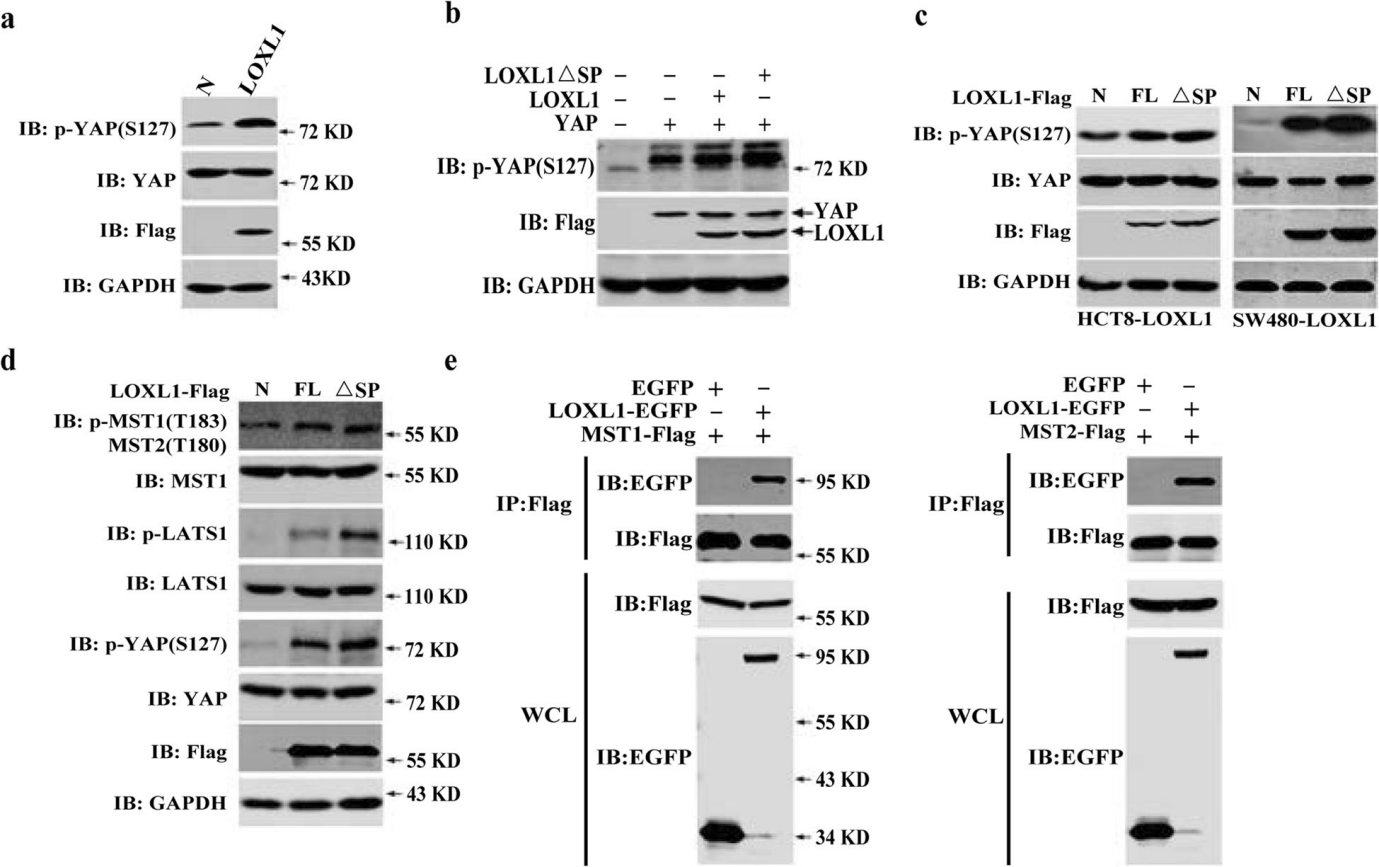
LOXL1 activates the Hippo pathway through interaction with MST1/2. **a** LOXL1 and empty vector were expressed in HEK293T cells for 24 h. Cell lysates were analysed by immunoblotting with the indicated antibodies. **b** LOXL1, LOXL1 △SP and YAP alone or combined as indicated were transfected into HEK293T cells. The cellular extracts were immunoblotted with the indicated antibodies. **c** LOXL1 and LOXL1 △SP were transfected into HCT8/SW480 cells for 24 h. Cell lysates were analysed by immunoblotting with the indicated antibodies. **d** LOXL1 and LOXL1 △SP were transfected into HEK293T cells for 24 h. The cell lysates were analysed using the indicated antibodies. **e** EGFP or LOXL1-EGFP was cotransfected with MST1-Flag or MST2-Flag in HEK293T cells and subjected to immunoprecipitation with anti-Flag M2 magnetic beads. The immunoprecipitated complexes (upper panels) and whole cell lysates (lower panels) were immunoblotted with the indicated antibodies

YAP is regulated by a myriad of extrinsic and intrinsic signals, including soluble extracellular factors, stress signals, cell-cell contact, mechanotransduction, and cell polarity [8]. These signals mainly regulate the phosphorylation events of the core MST–LATS kinase cascade and lead to the phosphorylation of YAP. Here, we investigated the mechanism by which LOXL1 and LOXL1 ∆SP decreases the activity but not the expression of YAP. We speculated that LOXL1 affects the phosphorylation of YAP. Interestingly, western blot analysis revealed that the level of p-YAP (S127) was increased significantly in HEK293T cells overexpressing LOXL1 (Fig. 5a), and LOXL1 silencing significantly reduced the phosphorylation levels of MST1/2 and YAP compared with those in the negative control in HT29 cells (Fig. S4). Simultaneously, the phosphorylation of p-YAP (S127) was found to be elevated with LOXL1 ∆SP compared to LOXL1 FL after their transient expression in HEK293T and CRC cells (HCT8 and SW480, Fig. 5b and c). We further detected the activities of the MST-LATS kinase cascade, which was mainly upstream. The results showed that the overexpression of LOXL1 and LOXL1 ∆SP in HEK293T cells activated the MST-LATS kinase cascade. Compared to LOXL1 FL, intracellular LOXL1 has a much better ability to activate the Hippo signalling pathway (Fig. 5d). The activation of MST1/2 kinases is considered to be the initial event in Hippo signalling [33]. Co-immunoprecipitation studies have shown that LOXL1 can interact with MST1 and MST2 (Fig. 5e), based on which we elucidated the effect of LOXL1 on the activation of MST kinase. These results indicated that intracellular LOXL1 can interact with MST kinase and then activate it to restrain the transcriptional activity of YAP.

### LOXL1 △SP inhibits the migration and invasion of CRC cells more potently than LOXL1 FL

**Fig. 6:**
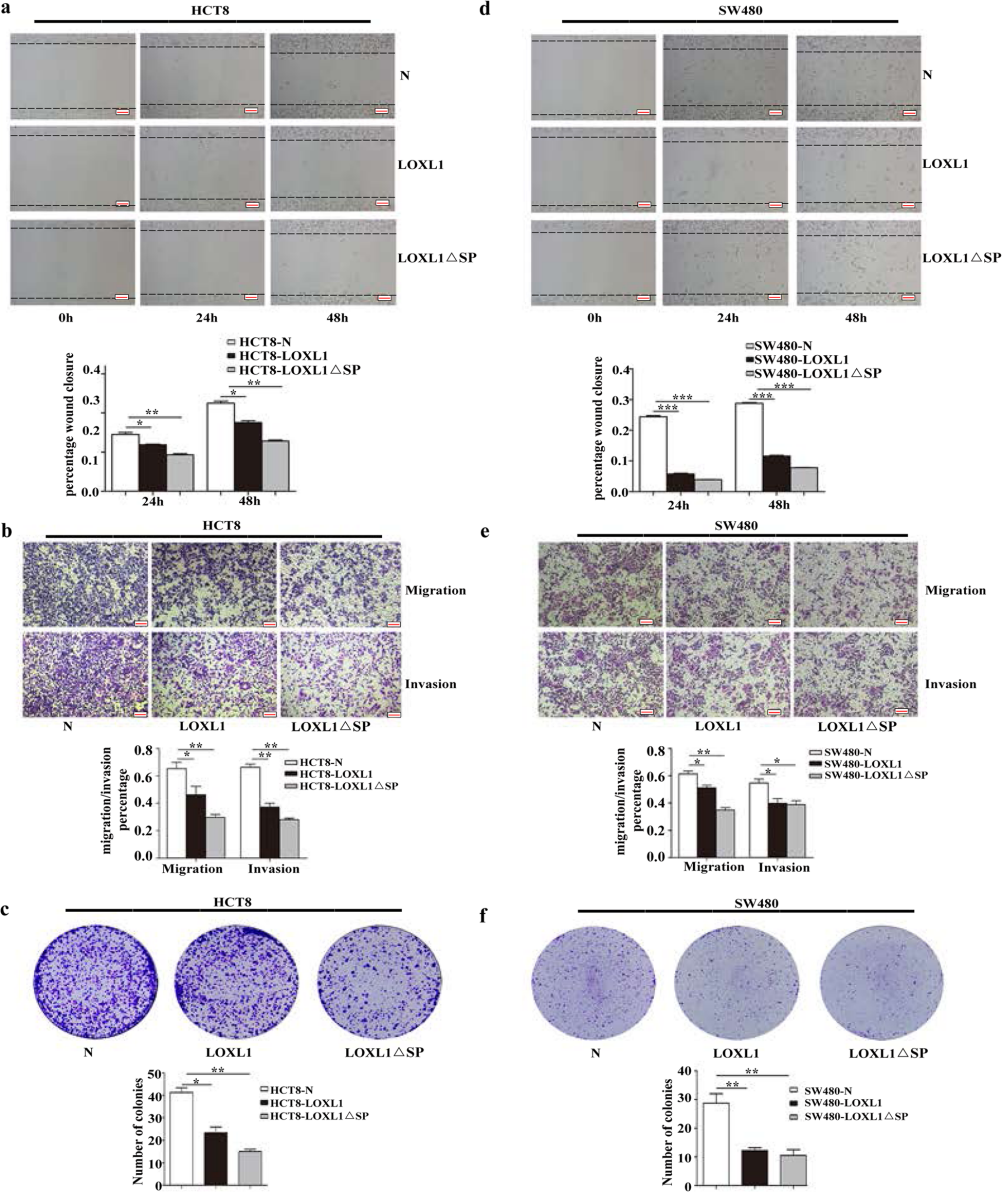
Effect of LOXL1 △SP expression on the migration and tumorigenesis of HCT8 and SW480 cells in vitro*.* **a** Wound healing analysis carried out in HCT8 cells transfected with the control vector, LOXL1 and LOXL1 △SP expression vectors at 0 h, 24 h, and 48 h (upper panel), and the calculation of their wound healing percentages (lower panel). Data are shown as the mean ± SD of triplicate independent sets of experiments; statistical significance was assessed by unpaired t-test. *P < 0.05, **P < 0.01. **b** Transwell migration and Matrigel invasion assays conducted using the overexpressed cell lines HCT8-N, HCT8-LOXL1 and HCT8-LOXL1 △SP (upper panel) and calculation of the rate of migration/invasion in relevant stable HCT8 cell lines (lower panel). Data are shown as the mean ± SD of triplicate independent sets of experiments; statistical significance was assessed by unpaired t-test. *P < 0.05, **P < 0.01. **c** A colony formation assay was performed using HCT8 cells after transfection with LOXL1 or LOXL1 △SP. An empty vector was used as the negative control. Upper panel: representative images, lower panel: quantification analysis. Data are shown as the mean ± SD of triplicate independent sets of experiments; statistical significance was assessed by unpaired t-test. *P < 0.05, **P < 0.01. **d** Wound healing analysis carried out on SW480 cells transfected with the LOXL1 expression vector, control vector, and LOXL1 △SP vector at 0 h, 24 h, and 48 h (upper panel), and calculation of their wound healing percentages (lower panel). Data are shown as the mean ± SD of triplicate independent sets of experiments; statistical significance was assessed by unpaired t-test. ***P < 0.001. **e** Transwell migration and Matrigel invasion assays using the overexpression cell lines SW480-N, SW480-LOXL1, SW480-LOXL1 △SP (upper panel) and calculation of the rate of migration/invasion for relevant SW480 cell lines (lower panel). Data are shown as the mean ± SD of triplicate independent sets of experiments; statistical significance was assessed by unpaired t-test. *P < 0.05, ***P < 0.01. **f** A colony formation assay was carried out using SW480 cells after transfection with LOXL1 or LOXL1 △SP. An empty vector was used as the negative control. Upper panel: representative images, lower panel: quantification analysis. Data from independent experiments are presented as the mean ± SD; statistical significance was assessed by unpaired t-test. **P < 0.01

LOXL1 is a secreted lysine oxidase that can be cleaved by morphogenetic protein-1 (BMP-1), leading to enzyme activation [34]. Three variants of LOXL1 are known, and we found that intracellular LOXL1 functions as an MST kinase activator. To further explore the criticality of the role of intracellular LOXL1 in CRC, we ectopically expressed N/LOXL1/LOXL1 ∆SP in HCT8 and SW480 cells using lentiviral constructs. Wound healing and Transwell assays were performed using HCT8 and SW480 cells to investigate the effect of stable overexpression of LOXL1 and LOXL1 ∆SP on cell migration and invasion abilities. The results showed that compared to LOXL1, LOXL1 ∆SP significantly decreased the migration ability of HCT8 and SW480 cells (Fig. 6a, b, d and e).

To assess whether YAP is an effective target of LOXL1, LOXL1 and YAP were co-transfected into HCT8 cells. The results showed that the co-expression of YAP was sufficient to cancel the inhibitory effect of LOXL1 in Transwell migration and Matrigel invasion assays. (Fig. S5) The results of colony formation assays revealed that in HCT8 and SW480 cells, the overexpression of LOXL1 resulted in the significant inhibition of colony formation ability compared to that observed in the control cells, while expression LOXL1 ∆SP had a stronger inhibitory effect than LOXL1 FL (Fig. 6c and f). These observations suggested that LOXL1 acts as a negative regulator of migration, invasion, and tumorigenesis by inhibiting YAP activity in CRC cells. We also found that LOXL1 ∆SP suppressed the secretion of LOXL1 and this truncation played a major role in inhibiting the malignant progression of CRC.

### Overexpression of LOXL1 inhibits tumorigenesis in vivo

**Fig. 7:**
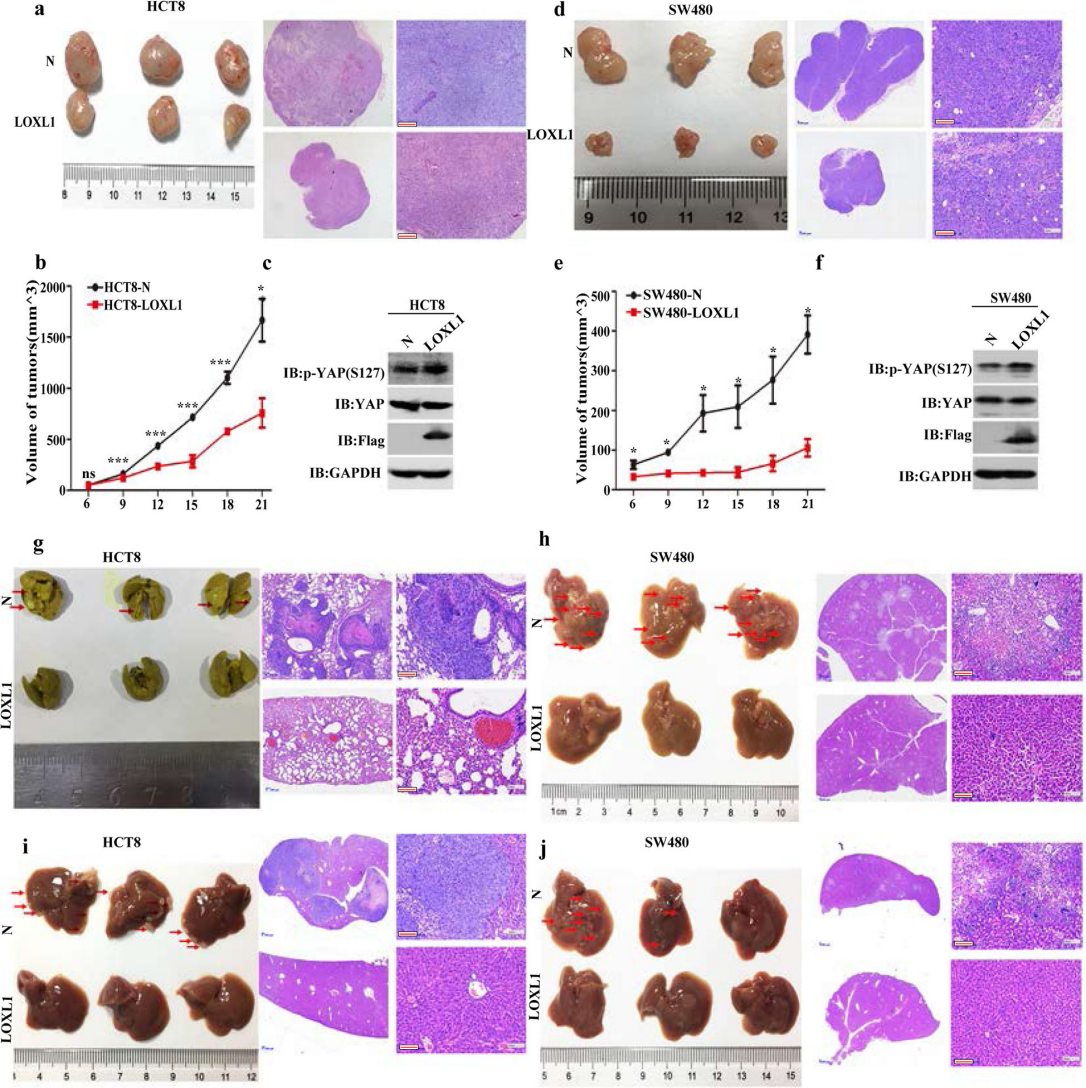
Effect of LOXL1 overexpression on CRC tumorigenesis in vivo*.* **a** Left: images of the xenograft tumours of HCT8-N and HCT8-LOXL1 in 4-week-old male nude BALB/c mice, right: corresponding images of haematoxylin-eosin (H-E) staining at magnifications of 25× and 100×. Scale bar: 100 μm. **b** Growth curve analyses of tumour volumes in HCT8-N/HCT8-LOXL1 cells measured from day 7 to day 19. Tumour width and length were measured with callipers, and growth curves were plotted based on the mean tumour volume at the indicated time points. Data are represented as the mean ± SD; *P < 0.05, ***P < 0.001, *n* = 3. **c** Protein extraction from HCT8-N/HCT8-LOXL1 xenograft tumours was immunoblotted with p-YAP (S127), YAP, Flag tag and GAPDH antibodies. **d** Left: images of xenograft tumours of SW480-N and SW480-LOXL1 in 4-week-old male nude BALB/c mice, right: corresponding images of H-E staining at magnifications of 25× and 100×. Scale bar: 100 μm. **e** Growth curve analyses of tumour volumes in SW480-N/SW480-LOXL1 cells measured from day 7 to day 19. Tumour width and length were measured with callipers, and growth curves were plotted based on mean tumour volume at the indicated time points. Data are represented as the mean ± SD; *P < 0.05, *n* = 3. **f** Protein extracts from SW480-N/SW480-LOXL1 xenograft tumours were immunoblotted with the indicated antibodies. **g** Left: corresponding images of the lungs of nude BALB/c mice after injection of HCT8-N and HCT8-LOXL1 cells into their tail veins for 8 weeks. (*n* = 3 mice/group); right: representative H-E images of metastases observed in the lungs of mice. Scale bar: 100 μm. **h** Left: SW480-N and SW480-LOXL1 cells were injected into the tail vein of nude BALB/c mice and relevant images of metastatic tumours in their livers (*n* = 3 mice/group); right: representative H-E image of metastases observed in the livers of mice. Scale bar: 100 μm. **i-j** left: HCT8-N/HCT8-LOXL1 and SW480-N/SW480-LOXL1 cells were injected into the spleens, and metastatic tumours in the livers were assessed 20 days after injection. The relevant images of mouse livers were captured (*n* = 3 mice/group); right: representative H-E image of metastases observed in the livers of mice. Scale bar: 100 μm

To further explore the effect of LOXL1 on tumorigenesis in vivo, cells overexpressing LOXL1 (HCT8-LOXL1, SW480-LOXL1) and their corresponding controls (HCT8-N, SW480-N) were subcutaneously injected into nude mice in the form of xenografts. The ectopic expression of LOXL1 was found to significantly decrease the size of the xenograft tumours in mice injected with HCT8 and SW480 cells, and corresponding results were observed upon haematoxylin-eosin (H-E) staining (Fig. 7a and d). To test the cell proliferation and YAP activity in xenografted tumours, p-YAP (S127) and Ki 67 were detected by immunohistochemistry (Fig. S6 b and c). Cell apoptosis in xenografted tumours was also measured by the TUNEL assay (Fig. S6d). Immunohistochemical analysis showed increased staining for p-YAP (S127) in LOXL1 overexpressing xenograft tumours compared with the control tumours. However, there was no significant change in the level of Ki 67 and TUNEL staining. Furthermore, tumour growth was also inhibited by the overexpression of LOXL1, as observed from the tumour growth curve (Fig. 7b and e). Western blot analysis showed that the overexpression of LOXL1 increased the expression of p-YAP (S127; the content of total YAP had not changed; Fig. 7c and f).

To explore if LOXL1 could promote the metastasis of CRC cells in vivo, cells overexpressing LOXL1 (HCT8-LOXL1, SW480-LOXL1) and their corresponding controls (HCT8-N, SW480-N) were injected into the lateral tail veins of nude mice. The results were observed after 8 weeks and revealed that compared to the control vector-containing HCT8 cells, LOXL1-overexpressing HCT8 cells had repressed tumour metastasis in the lungs of mice. Picric acid was used to visualize and fix the samples of mouse lung to observe the metastases (Fig. 7g). However, no metastases were observed in the lungs of mice injected with the SW480 cell lines, but metastases were present in the livers of mice injected with the SW480-N cells. No metastases were observed in the SW480-LOXL1 group (Fig. 7h).

We further investigated the functional relevance of LOXL1 with the metastasis observed in the liver. HCT8-LOXL1, SW480-LOXL1 and their corresponding control cells were slowly injected into the spleen; the overexpression of LOXL1 drastically decreased the number and size of metastatic tumours in the livers of mice (Fig. 7i and j). Collectively, the in vivo results demonstrated the criticality of the role of LOXL1 as a tumour suppressor in the metastasis of CRC cells.

## Discussion

Increasing evidence has revealed that the lysyl oxidase, LOXL1 is involved in the malignant progression of cancer [18, 23, 35]. However, the underlying molecular role of LOXL1 in CRC has not been elucidated. Here, we aimed to evaluate the molecular mechanisms involved in LOXL1-mediated cell migration, invasion, and tumorigenesis in CRC. Our present study has revealed novel mechanisms through which LOXL1 was found to suppress the metastasis of CRC. We detected that the overexpression of LOXL1 inhibited the migration, invasion, and tumorigenesis of CRC cells in vivo and in vitro, whereas opposite effects were observed upon its downregulation. Previous studies have reported that LOX is a tumour suppressor, the expression of which was found to be reduced in the tumour tissue, and its downregulation was controlled through methylation [36, 37]. However, evidence has indicated that the tumour suppressor activity of LOX is dependent on the pro-peptide domain and not the enzyme catalytic domain [38–41]. Since LOXL1 is highly homologous to LOX, we hypothesized that its tumour suppressor activity is also dependent on its intracellular function. Our results proved that when the SP was deleted from LOXL1 (LOXL1 ∆SP), the extracellular secretion of LOXL1 was inhibited. Furthermore, the suppression of tumour metastasis was more pronounced after the cytoplasmic retention of LOXL1 ∆SP than that observed with LOXL1 in vitro.

Previous studies have reported that YAP can regulate the expression of members belonging to the LOX family [42–44]. We found that LOXL1 was involved in the progression of the Hippo pathway. The core kinase signalling cassette and components of the Hippo pathway are highly conserved [45]. Overall, activated MST1/2 interacts with SAV1 through the SARAH domains, leading to phosphorylation and activation of LATS1/2, which suppresses the carcinogenicity of YAP by promoting its phosphorylation at Ser 127 and its cytoplasmic retention [46]. As a central component of the Hippo signalling pathway, the critical role of YAP has been widely reported in CRC. However, the modulators of YAP have not been well described previously [6, 7, 47]. We identified for the first time that LOXL1 is a novel regulator of YAP involved in CRC tumorigenesis. In our study, we revealed that LOXL1 could inhibit the malignant progression of cells by inducing the activity of MST kinase, which leads to the inhibition of the transcriptional activity of YAP in CRC. Consistent with our hypothesis, we observed that the LOXL1 enzyme mutants also inhibited the transcriptional activity of YAP, which may have occurred due to the interaction of intracellular LOXL1 with MST kinase. Numerous upstream components have been identified to modulate the kinase activity of MST1/2, including mechanotransduction, cell polarity, and G-protein-coupled receptor (GPCR) signalling [45]. MST1/MST2 have an N-terminal catalytic domain and a C-terminal SARAH domain. The SARAH domain-containing coiled-coil domain mediates self-association as well as association with other SARAH domain-containing proteins to regulate MST1/2 kinase activity [48, 49]. The N-terminal region of LOXL1 is thought to be important in protein-protein interactions. We assume that LOXL1, as a scaffold protein, directly or indirectly interacts with MST1/MST2 to promote the dimerization of MST1/MST2, thereby promoting the kinase activity of MST1/2. However, the molecular mechanisms through which LOXL1, particularly intracellular LOXL1, activates the Hippo signalling pathway are still unclear and need to be studied further.

Furthermore, our results have established the major role played by LOXL1 in the molecular mechanism of CRC development, since it was found to inhibit the transcription of *YAP*, a classical gene involved in the Hippo signalling pathway, to inhibit the development of CRC. It is well known that tumours are developed as a result of multi-gene, multi-stage altering processes, and emerging evidence has suggested the involvement of numerous oncogenes in the process of CRC tumourigenesis and malignant progression. However, little information is available on the role of tumour suppressor genes associated with CRC. Based on our findings, we believe that tumour suppressor genes, such as *LOXL1*, could provide potential drug targets for intervening in the malignant progression of CRC. We have revealed that LOXL1 can inhibit the development of CRC by inhibiting *YAP* gene transcription.

## Conclusions

In conclusion, our results revealed evidence about the contributions of LOXL1 in inhibiting the malignant progression of CRC, including its suppression migration, invasion and proliferation. Taken together, our studies encourage further efforts to uncover and evaluate LOXL1 related drug targets mediating the malignant progression of CRC and provide molecular mechanisms to support a new theoretical basis for the advancement of clinical treatments.

## Supplementary information

**Additional file 1: Figure S1.** LOXL1 overexpression inhibits the proliferation of CRC cells. a HCT8-N/HCT8-LOXL1 and SW480-N/SW480-LOXL1 cells were detected by CCK8 analysis. Data are shown as the mean ± SD of triplicate independent sets of experiments; statistical significance was assessed by unpaired t-test. ***P < 0.001, ****P < 0.0001. b Apoptosis was analysed by *7AAD/Annexin-V* labeling. Representative dot plots shown on the left, quantified for apoptosis on right. Data are shown as the mean ± SD of triplicate independent sets of experiments; statistical significance was assessed by unpaired t-test. ns; non-significant.

**Additional file 2: Figure S2.** Knockdown of LOXL1 in RKO and HT29 cells increases their proliferation ability in vitro. CCK8 analysis was performed to detect the cell proliferation. Data from three independent experiments are presented as the mean ± SD. Statistical significance was assessed by unpaired t-test; ***P < 0.001, ****P < 0.0001.

**Additional file 3: Figure S3.** LOXL1 negatively regulates the YAP activity. a LOXL1 and YAP constructs alone or combined, as indicated, were transfected into HEK293T cells together with a indicated luciferase reporters. The results shown were normalized for transfection efficiency. Data are shown as the mean ± SD of triplicate independent sets of experiments; statistical significance was assessed by unpaired t-test. ns; non-significant, ***P < 0.001. b Immunofluorescence to detect the localization of endogenous YAP by overexpression of LOXL1-EGFP in HCT8 cells. Scale bar: 20 μm.

**Additional file 4: Figure S4** Knockdown of *LOXL1* decreases the Hippo pathway activation. HT29 cells were transfected with *siRNA* to LOXL1 or a control *siRNA* (N) for 48 h. Cell lysates were analysed by immunoblotting with the indicated antibodies.

**Additional file 5: Figure S5.** YAP can reverse the inhibitory effect of LOXL1. Transwell migration and Matrigel invasion assays were performed in LOXL1 alone or LOXL1 and YAP co-transfected HCT8 cells. Representative images (left panel) and quantification (right panel) are shown as indicated. Data from three independent experiments are presented as the mean ± SD. Statistical significance was assessed by unpaired t-test; **P < 0.01. Scale bar: 100 μm.

**Additional file 6: Figure S6.** a H-E staining of HCT8-N/HCT8-LOXL1 and SW480-N/SW480-LOXL1 xenograft tumours. b and c HCT8-N/HCT8-LOXL1 and SW480-N/SW480-LOXL1 xenograft tumours were detected the p-YAP(S127) and Ki67 levels by immunohistochemistry. d Cell apoptosis in xenografted tumors was measured by the TUNEL assay. Scale bar: 100 μm.

## Data Availability

Source data and reagents are available from the corresponding author upon reasonable request.

## Abbreviations

CRC: Colorectal cancer
LOXL1: Lysyl oxidase-like 1
IHC: Immunohistochemistry
HE: Haematoxylin and eosin
IP: Immunoprecipitation
