# Supplementary figures and images for: LOXL1 modulates the malignant progression of colorectal cancer by inhibiting the transcriptional activity of YAP
Source: Cell Commun Signal. 2020 Sep 10;18:148. doi: 10.1186/s12964-020-00639-1 (PMC7488294; doi:10.1186/s12964-020-00639-1)

**a**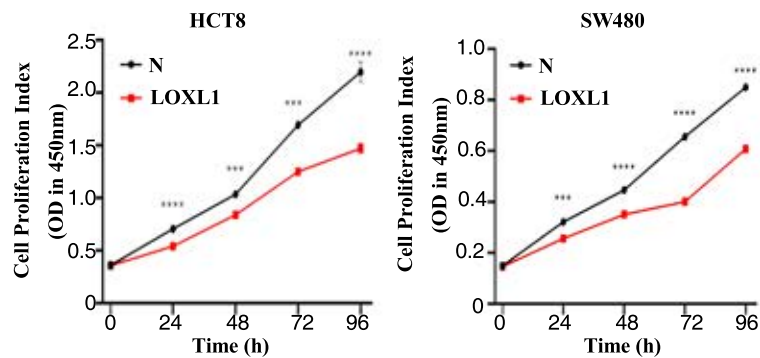**b**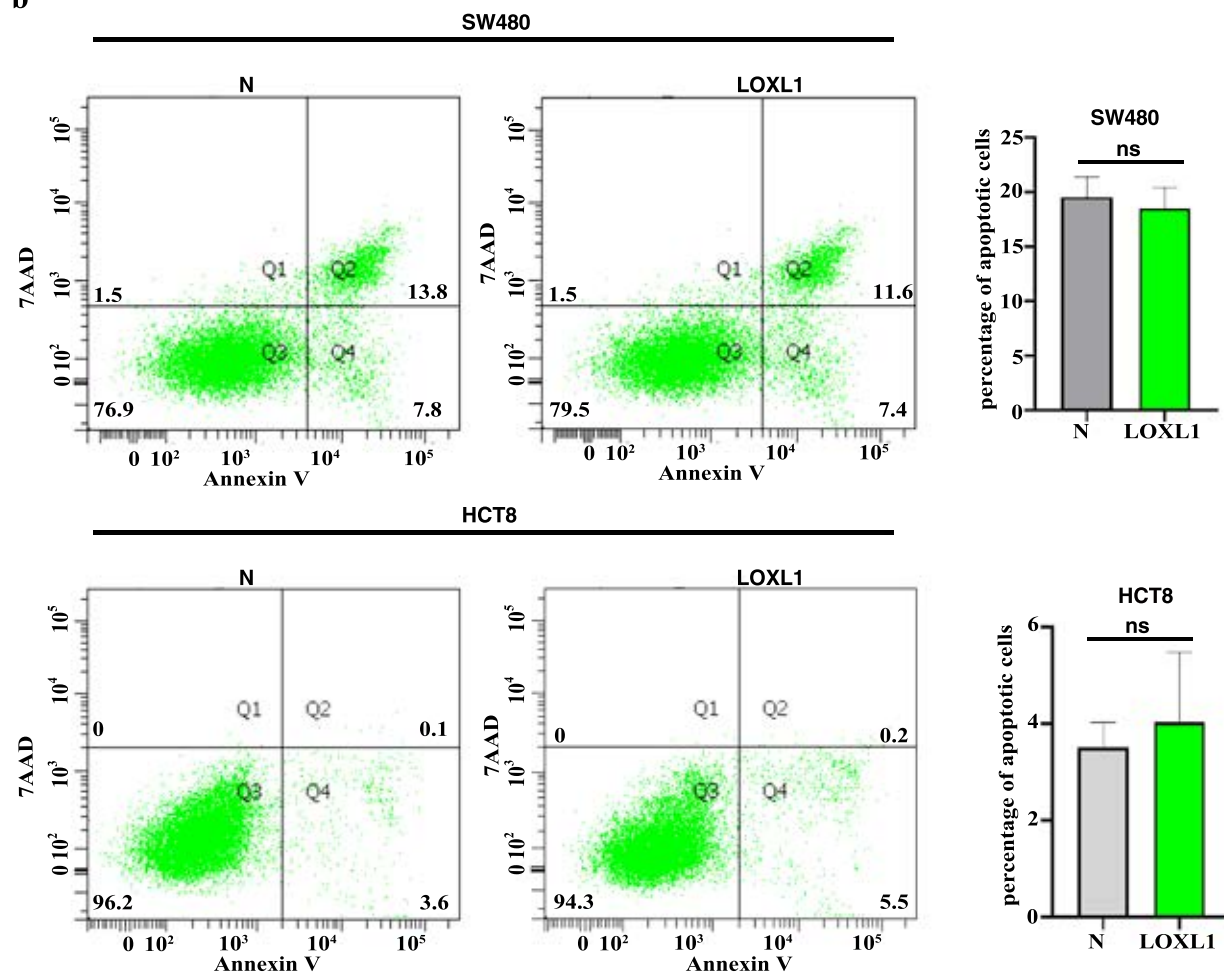

Supplement: Supplementary file 2 — Additional file 1: Figure S1. LOXL1 overexpression inhibits the proliferation of CRC cells. a HCT8-N/HCT8-LOXL1 and SW480-N/SW480-LOXL1 cells were detected by CCK8 analysis. Data are shown as the mean ± SD of triplicate independent sets of experiments; statistical significance was assessed by unpaired t-test. ***P < 0.001, ****P < 0.0001. b Apoptosis was analysed by 7AAD/Annexin-V labeling. Representative dot plots shown on the left, quantified for apoptosis on right. Data are shown as the mean ± SD of triplicate independent sets of experiments; statistical significance was assessed by unpaired t-test. ns; non-significant. [file 12964_2020_639_MOESM2_ESM.pdf]

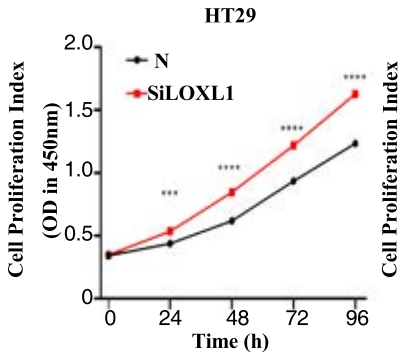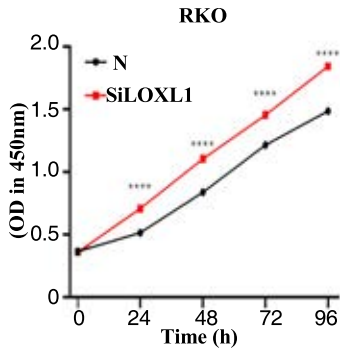

Supplement: Supplementary file 3 — Additional file 2: Figure S2. Knockdown of LOXL1 in RKO and HT29 cells increases their proliferation ability in vitro. CCK8 analysis was performed to detect the cell proliferation. Data from three independent experiments are presented as the mean ± SD. Statistical significance was assessed by unpaired t-test; ***P < 0.001, ****P < 0.0001. [file 12964_2020_639_MOESM3_ESM.pdf]

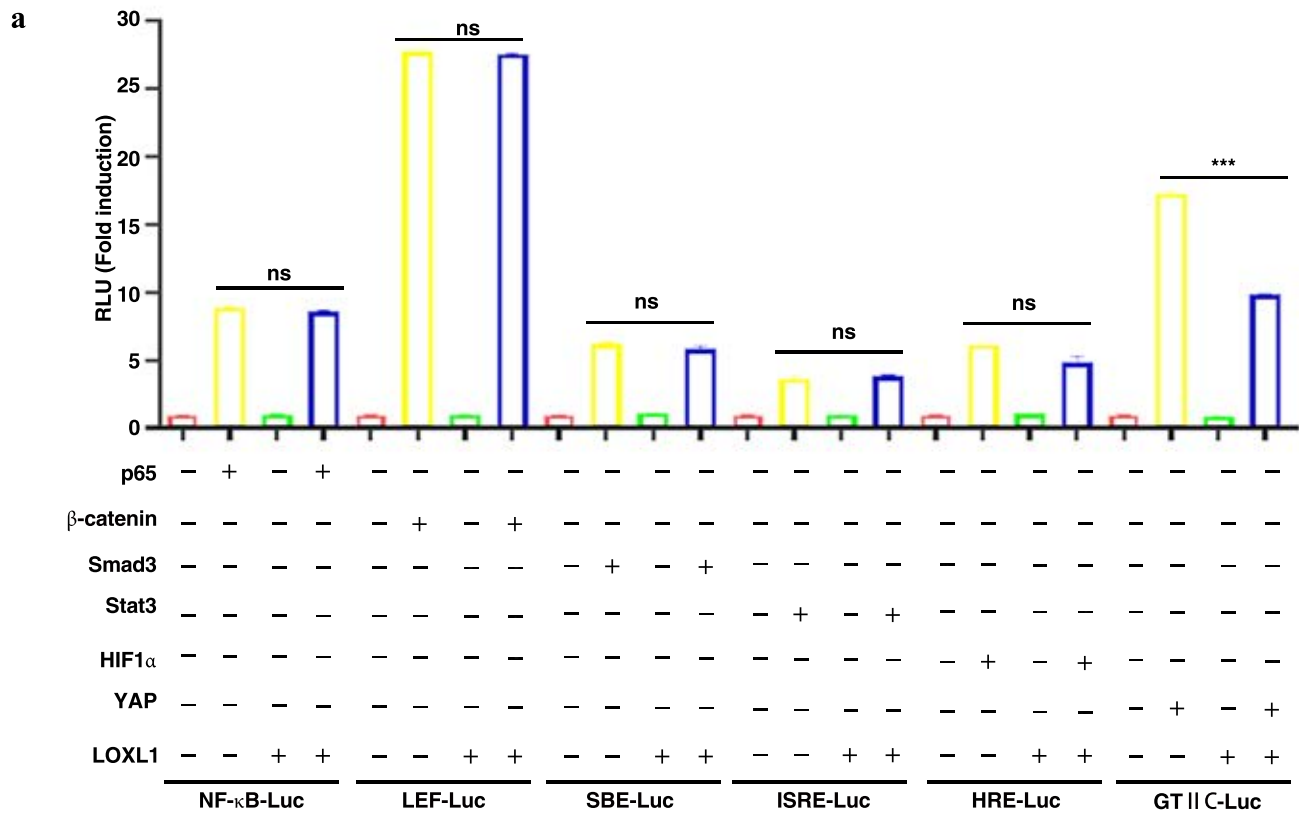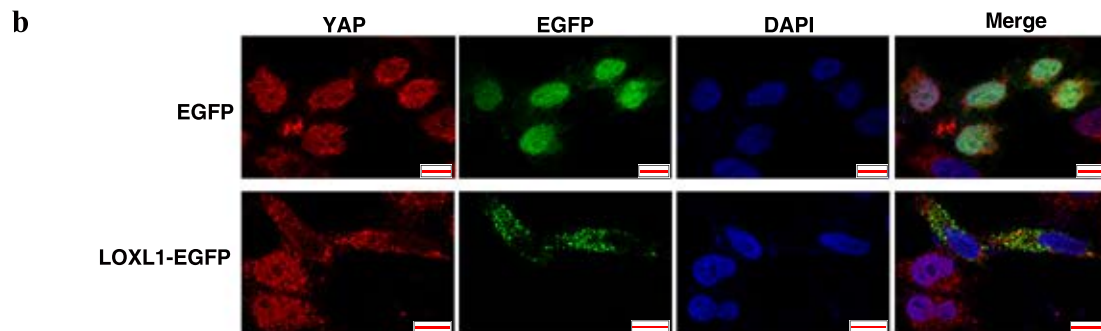

Supplement: Supplementary file 4 — Additional file 3: Figure S3. LOXL1 negatively regulates the YAP activity. a LOXL1 and YAP constructs alone or combined, as indicated, were transfected into HEK293T cells together with a indicated luciferase reporters. The results shown were normalized for transfection efficiency. Data are shown as the mean ± SD of triplicate independent sets of experiments; statistical significance was assessed by unpaired t-test. ns; non-significant, ***P < 0.001. b Immunofluorescence to detect the localization of endogenous YAP by overexpression of LOXL1-EGFP in HCT8 cells. Scale bar: 20 μm. [file 12964_2020_639_MOESM4_ESM.pdf]

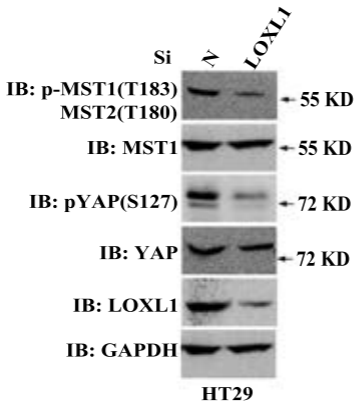

Supplement: Supplementary file 5 — Additional file 4: Figure S4 Knockdown of LOXL1 decreases the Hippo pathway activation. HT29 cells were transfected with siRNA to LOXL1 or a control siRNA (N) for 48 h. Cell lysates were analysed by immunoblotting with the indicated antibodies. [file 12964_2020_639_MOESM5_ESM.pdf]

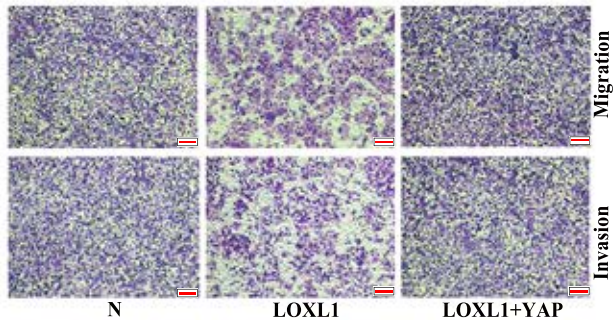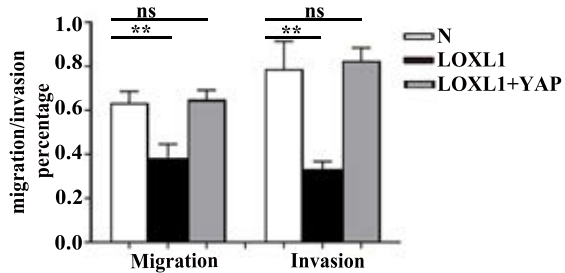

Supplement: Supplementary file 6 — Additional file 5: Figure S5. YAP can reverse the inhibitory effect of LOXL1. Transwell migration and Matrigel invasion assays were performed in LOXL1 alone or LOXL1 and YAP co-transfected HCT8 cells. Representative images (left panel) and quantification (right panel) are shown as indicated. Data from three independent experiments are presented as the mean ± SD. Statistical significance was assessed by unpaired t-test; **P < 0.01. Scale bar: 100 μm. [file 12964_2020_639_MOESM6_ESM.pdf]

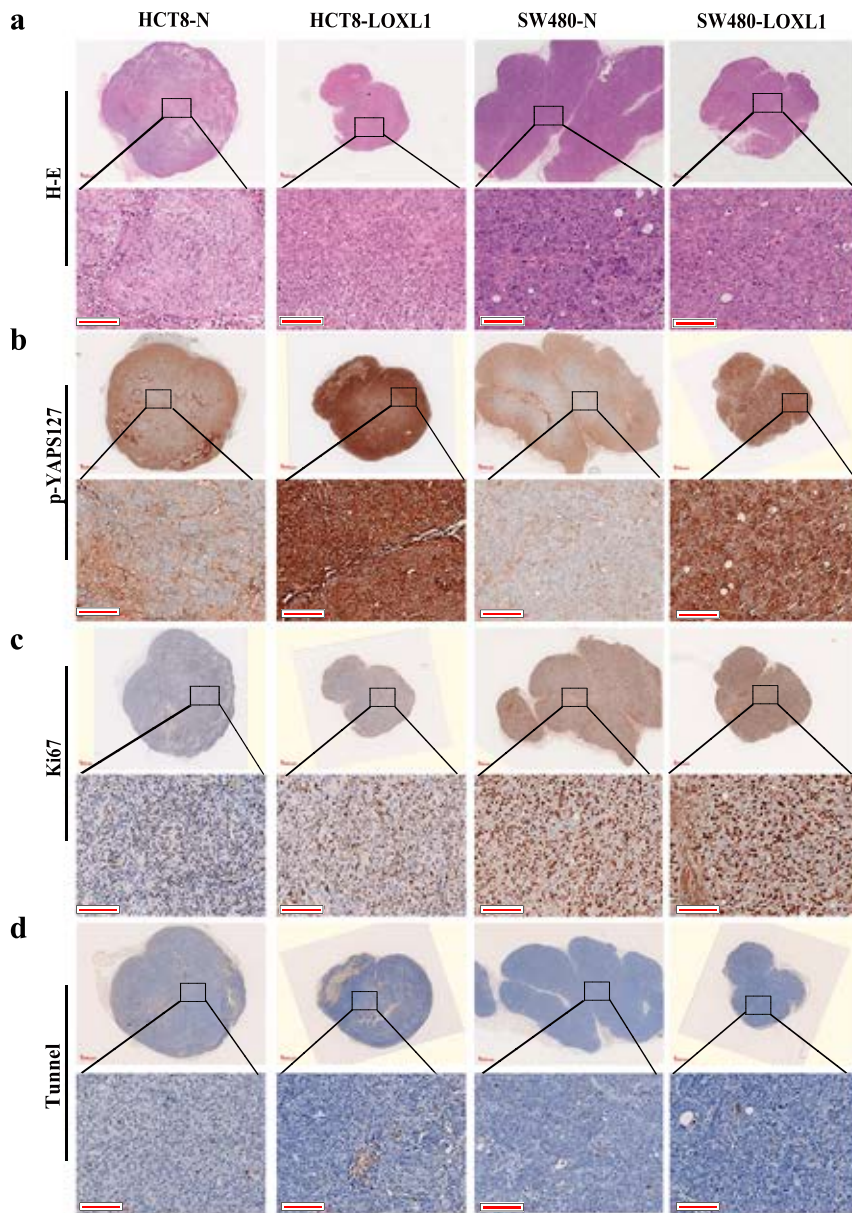

Supplement: Supplementary file 7 — Additional file 6: Figure S6. a H-E staining of HCT8-N/HCT8-LOXL1 and SW480-N/SW480-LOXL1 xenograft tumours. b and c HCT8-N/HCT8-LOXL1 and SW480-N/SW480-LOXL1 xenograft tumours were detected the p-YAP(S127) and Ki67 levels by immunohistochemistry. d Cell apoptosis in xenografted tumors was measured by the TUNEL assay. Scale bar: 100 μm. [file 12964_2020_639_MOESM7_ESM.pdf]
